# Effectiveness of an improved road safety policy in Ethiopia: an interrupted time series study

**DOI:** 10.1186/1471-2458-14-539

**Published:** 2014-05-31

**Authors:** Teferi Abegaz, Yemane Berhane, Alemayehu Worku, Abebe Assrat

**Affiliations:** 1 School of Public and Environmental Health, College of Medicine and Health Sciences, Hawassa University, Hawassa, Ethiopia; 2 Addis Continental Institute of Public Health, Addis Ababa, Ethiopia; 3 School of Public Health, College of Health Sciences, Addis Ababa University, Addis Ababa, Ethiopia; 4 Ministry of Transport, Addis Ababa, Ethiopia

**Keywords:** Road crash, Road injury, Road death, Road safety law effectiveness

## Abstract

**Background:**

In recent years, there has been an increasing interest in implementing road safety policy by different low income countries. However; the evidence is scarce on its success in the reduction of crashes, injuries and deaths. This study was conducted to assess whether road crashes, injuries and fatalities was reduced following the road safety regulation introduced as of September 2007 by Oromia Regional State Transport Bureau.

**Methods:**

Routine road traffic accident data for the year 2002-2011were collected from sixteen traffic police offices. Data on average daily vehicle flow was obtained from the Ethiopian Road Authority. Interrupted time series design using segmented linear regression model was applied to estimate the effect of an improved road safety policy.

**Results:**

A total of 4,053 crashes occurred on Addis Ababa - Adama/Hawassa main road. Of these crashes, almost half 46.4% (1,880) were property damage, 29.4% (1,193) were fatal and 24.2% (980) injury crashes, resulting 1,392 fatalities and 1,749 injuries. There were statistically significant reductions in non-injury crashes and deaths. Non-injury crash was reduced by 19% and fatality by 12.4% in the first year of implementing the revised transport safety regulation.

**Conclusion:**

Although revised road safety policy helped in reducing motor vehicle crashes and associated fatalities, the overall incidence rate is still very high. Further action is required to avoid unnecessary loss of lives.

## Background

Road traffic crash is a growing public health threat, being responsible for 1.2 million deaths and up to 50 million nonfatal injuries globally. It is a big challenge especially for low and middle income countries, 90% of the victims were found [1]. Accident trends observed in industrialized countries witnessed the rapid reduction over the last three to four decades, while a terrifying increment reported from a number of developing countries including Ethiopia [2,3]. The problem may grow further in the coming decades; due to the rapid rising of vehicle ownership associated with their economic growth [4].

Evidence from high income countries showed that implementation of appropriately designed and well enforced road safety policy contribute a lion share for their impressive achievements of the declining trend in the number and severity of crashes [5]. According to the European Transport Safety Council estimation, if all existing road safety laws in European Union are enforced up to 50% of death and injuries could be averted [6].

Likewise in recent years, many low income countries are implementing improved road safety policy to deter risky driving practices including: - exceeding the speed limit, impaired driving by alcohol and drugs, phoning and texting while driving, and not using seat belt [5,7]. However there is little empirical evidence as to their effectiveness in reducing crashes, injuries and fatalities [7]. For example, Brazil, after implementing an improved traffic code with stiff penalty and media coverage a 21% and 25% reduction of injuries and fatalities, respectively, was observed [8]. A study conducted in Uganda following, police enforcement using patrol cars equipped with radar brings a 17% reduction of fatalities [9]. In Rwanda a 30% reduction in traffic death was observed following an improved legislative change complimented with public awareness campaign [10].

This study was conducted to measure the level of effectiveness of an improved road safety policy (Oromia Regional State Road Transport Regulation No; 96/2007) enforced as of September 2007. The Oromia Regional state is one of the largest states in Ethiopia. This improved road safety policy include the new road safety laws (prohibition of cell phone conversation while behind the wheel, driving without using a seat belt and not using motorcycle helmet) and the amendment of the existing road safety laws (excessive speeding, impaired driving with alcohol and Khat and unsafe loading) by introducing higher penalty rate including suspension of the drivers’ licens. Seat balt wearing, healmet use and phoning while driving were enforced by using a roadside random check up on a regular bases; however speed and alcohol were not well enforced due to lack of radar and breath analyzer.

## Methods

### Study design

Interrupted time series design was utilized to evaluate the effectiveness of an improved road safety policy implemented by Oromia Regional State Transport Bureau. Interrupted time series design is an alternative approach used to evaluate the effects of any intervention, when randomized control trials are infeasible or identification of a control group impractical [11]. This design was utilized by various researchers to assess the effectiveness of health care intervention [11,12].

### Study setting

The study was conducted on one of the main and busiest roads of Ethiopia, which extends south from the capital Addis Ababa to Adama/Hawassa. This two-way and two-lane road has an average width of 8 meters and covers a total distance of 264 Km. It is part of the main route of the country’s import and export corridor from the port of Djibouti and part of the Trans-African Highway (an international road that stretched from Cairo to Cape Town). Moreover, the road has a significant economic importance since many of the cash crops, floriculture farms, recreational areas and tourist centers are located across the stretches of the road. According to the Ethiopian Road Authority report more than 20, 000 vehicles used the road daily.

### Data sources

We reviewed traffic crash records, routinely collected by the police officers from 16 district traffic offices for the period 2002 through 2011. Additional data on daily vehicle flow was obtained from the Ethiopian Road Authority. Only crashes happened on Addis Ababa- Adama/Hawassa highway was considered for this study. A data retrieving form was developed and used to record information from the crash registration (CR) book. A total of 16 traffic officers, who are assigned as an expert in documentation and reporting of crash related cases were recruited from each of the 16 traffic offices and provide training. Furthermore, two senior police officers and the principal investigator were involved in the data collection process as a supervisor. Relevant information on the type and severity of the crash, type of vehicles and road users involved in the crash and the time and place of the crash was retrieved.

### Data management and quality assurance

Those crashes lacked some of the relevant information needed were excluded. Among the total 46 cases that excluded from the analysis, 27 of them were reported before the intervention and 19 of them reported after the intervention. The main reason for the exclusion is due to lack of information on injury severity and number of victims involved. The collected data was double entered by two different data clerk, using the EpiData 3.1 statistical software. Once the data entry completed, we run the frequency and print out the output of the two datasets. Consistency was checked by comparing the frequency and the difference was corrected accordingly by using the original data retrieving form.

### Statistical analysis

The revised road safety policy include the new road safety laws (banning of cell phone conversation, unbelted driving and not using motorcycle helmet) and the amendment of the existing road safety laws (excessive speeding, impaired driving with alcohol and Khat and unsafe loading) which was implemented as of September 2007. Prior to September 2007, road safety enforcement in Oromia Regional State implemented by police officer using less strict enforcement with low penalty. On the other hand, the existing and the newly enacted road safety laws were implemented in a coordinated way (traffic police and transport expert) using a stricter law enforcement to the extent of drivers’ licence suspension. Seat belt wearing, helmet use and unsafe loading were enforced by using a roadside random check up on a regular basis; however, lack of road safety instruments like; radar and breath analyzer hinder the implementation of speeding and alcohol intoxication.

In this study three different dependent variables were considered in the statistical analysis: monthly rates of non-injury crashes, fatalities and injuries per 10,000 vehicles. The explanatory variables were intervention dummy variable coded 0 before the intervention and coded 1 after the intervention. A time trend was used to control the confounding effect of the underlying trend on the actual intervention coded as 1 at the starting month of the first observation and continued to the last observation. To estimate the trend change we introduced a scaled interaction term (the number of months counted starting from the new policy intervention) time coded 1 at the start month and continued through the last month.

The monthly rate of non-injury crashes, fatalities and injuries were plotted over time and visually inspected to assess the trends or the non-stationarity of the data. We also used autocorrelation function (ACF) and partial autocorrelation function (PACF) and Dickey-Fuller unit root test to determine the nature of the trend. Both tests showed the trend was deterministic, hence segmented regression model (fit a least-squares regression line to each segment of the explanatory variables) was the recommended approach for model building process [13]. Autocorrelation was assessed by using Durbin-Watson statistics (for serial correlation) and Breusch and Godfrey (for higher order correlation). Durbin-Watson statistics showed serial correlation in one of the dependent variable (non-injury crashes). So fitting a least-squares regression line provide a biased estimate and hence adjustment was done by applying feasible GLS estimator (generalized least squares), using Prais-Winston method [14].

Model diagnostics were carried out using variance inflation factors and visual observation of the line graph to investigate the existence of collinearity and influential or outliers. Model fitness was checked by using change in percentile rank (CPR) statistics. Hence, only the trend change shows the statistical significant effect. Applying - R^2^ as model fitness was not applicable because it independently predicted by level change only (immediate effect after intervention) which is insignificant in all of the three models in our case.

This intervention study was performed using STATA 12 software package. Prais-Winston regression approach (model 1) was fitted for non-injury crashes and segmented regression using an ordinary least square approach (model 2 & 3) for both fatalities and injuries. Both models included 120 monthly observations. The effectiveness of the improved road safety intervention was assessed by using the formula indicated by equation (1) reference [11].

(1) $$\begin{array}{l} y_{t}=\beta_{0}+\beta_{1}*\mathrm{time}+\beta_{2}*\mathrm{inetervention}\\ \;+\beta_{3}*\mathrm{postslope}+\epsilon_{t} \end{array}$$

Where *y*_*t*_ is the outcome variable at time, time is a continuous variable indicating time (in months) at time *t* from the start (*t =* 0 month) until the end (*t =* 120 months) of the observation period (January 2002 to December 2011), intervention is an indicator variable for time *t* occurring before (*t =* 0 month) or after (*t =* 1 month) (September 2007) the change to an improved road safety policy; post-slope is coded 0 up to the last point before the intervention phase (January 2002 to August 2007) and (time 1–52) after the intervention of an improved road safety (September 2007 to December 2011) and *e*_*t*_ is the error term at time *t.* Similarly *β*_0_ value at time zero, *β*_1_ change over time before the intervention was implemented, *β*_2_ is change in the outcome measure from the last time point before the intervention to the first time point after the intervention and *β*_3_ difference in the slope of the time period before the intervention and the slope of the time period after the intervention.

In the absence of the improved road safety intervention the model predict the non-injury crashes, injuries and deaths given by equation (2) reference [11].

(2) $$y_{t}\mathrm{No}=\beta_{0}+\beta_{1}*\mathrm{time}$$

## Results

The result showed that, a total of 4,053 crashes occurred on the Addis Ababa - Adama/Hawassa main road during the study period. Almost half, 46.4% (1,880) were property damage only crashes; 29.4% (1,193) fatal crashes and the rest 24.2% (980) injury crashes. From 1,193 fatal crashes 1,392 people were dying, on average 1.2 deaths per fatal crashes. Of these deaths, more than half 57.5% (800) were pedestrian, 32% (445) vehicle occupants and 10.5% (147) drivers. During the 980 injury crashes 1,749 people were injured, on average, 1.8 injuries per crash, over half, 55.2% (965) were vehicle occupants, followed by pedestrian 35.1% (614) and the rest 9.7% (170) were drivers (Table 1).

**Table 1:** Base-line characteristics of non-injury crashes, deaths and injuries on the Addis Ababa - Adama/Hawassa main road from 2002-2011

**Characteristics**	**Number**	**Percentage (%)**
Severity of crashes (n = 4053)		
Property damage only crashes	1,880	46.4
Fatal crashes	1,193	29.4
Non-fatal crashes	980	24.2
Fatalities and injuries		
Fatalities (n = 1,392)		
Pedestrians	800	57.5
Vehicle occupants	445	32
Drivers	147	10.5
Injuries (n = 1,749)		
Pedestrians	614	35.1
Vehicle occupants	965	55.2
Drivers	170	9.7
Types of crashes		
Crash with other vehicles	1,645	40.6
Pedestrian collision	1,335	32.9
Rollover crash	651	16
Crash with fixed objects	238	6
Crash with animals and others	184	4.5
Time of the crash		
Day time	2,795	69
Night time	1,225	31

Regarding the type of crashes reported in the study area, 40.6% (1,645) were crashing with other vehicles, followed by pedestrian collision 32.9% (1,335), rollover crashes accounted 16% (651) and the rest 6% (238), 4.5% (184) crash with fixed object and others including animal vehicle crash respectively. Day time collision accounted 69% (2,795) of total crashes (Table 1).

**Figure 1:**
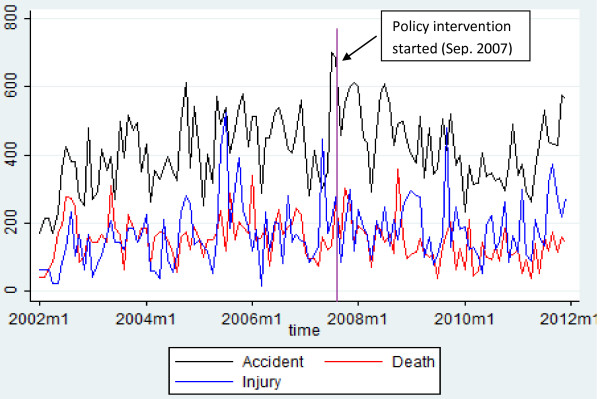
Monthly time series plot of non-injury crashes, deaths and injuries per 10,000 vehicles on the Addis Ababa - Adama/Hawassa main road from 2002–2011.

Figure 1 indicates the monthly time series trend of non-injury crashes, deaths and injuries before and after the intervention. The plot shows no observed seasonality and time trend before intervention. The monthly rates of non-injury crashes, deaths and injuries per 10,000 vehicles before and after the implementation of an improved road safety policy were illustrated in Table 2.

**Table 2:** Monthly rates of non-injury crashes, deaths and injuries per 10,000 vehicles before and after the implementation of an improved road safety policy on Addis Ababa-Adama/Hawassa road from 2002-2011

**Outcome of a crash**	**Monthly rates per 10,000 vehicles**	
	**Pre-intervention period (January 2002-Augest 2007)**	**Post-intervention period (September 2007-December 2011)**
Non-injury crashes	2.76	2.22
Deaths	1.10	0.70
Injuries	1.10	0.99

Table 3 summarizes the effect of the new improved road safety in non-injury crashes, injuries and deaths. According to a model 1 estimate, there were 295 non-injury crashes per 10,000 vehicles at the beginning of observation. Before and after intervention, the trend showed statically significant variation; increased by 3.2 (95% CI: 1.57 to 4.92) before and decreased by 5.1 (95% CI: -8.143365 to -2.04914) after intervention per 10,000 vehicles per month respectively. The estimated coefficients of the Model 1 showed that, after one year of intervention, we observed 451 crashes/10,000 vehicles (equation 1), however; in the absence of this intervention the model predicted an average of 558 per 10,000 vehicles per month (equation 2). Implies, the net effect of the intervention could show 101 non-injury crash reductions monthly per 10,000 vehicles or by 19.2%.

**Table 3:** The parameter estimates, standard errors, t statistics and confidence level of different models of non-injury crashes, deaths and injuries on the Addis Ababa - Adama/Hawassa main road from 2002-2011

**Models and variables**	**Coefficient**	**Standard error**	***t *** **statistics**	**Confidence interval**
Non-injury crashes: Prais-				
Winston regression (Model 1)				
Intercept β0	295.1577	33.8195	8.73***	(228.17, 362.14)
Baseline trend β1	3.248131	.8461874	3.84***	(1.57, 4.92)
Level change β2	-40.19842	49.2873	-.82	(-137.81, 57.42)
Trend change β3	-5.096253	1.538459	-3.31**	(-8.14, -2.05)
Death: Segmented regression (Model 2)				
Intercept β_0_	150.1638	15.06675	9.97***	(120.32, 1.10)
Baseline trend β_1_	.3492265	.3795861	.92	(-.40, 1.10)
Level change β_2_	3.599756	22.71791	.16	(-41.39, 48.59)
Trend change β_3_	-1.961251	.6828936	-2.87**	(-3.31, -.60)
Injuries: Segmented regression (Model 3)				
Intercept β_0_	96.99802	22.13401	4.38***	(53.16, 140.83)
Baseline trend β_1_	1.862395	.557636	3.34**	(.75, 2.96)
Level change β_2_	-43.5782	33.37405	-1.31	(-109.68, 22.52)
Trend change β_3_	-1.489333	1.003214	-1.48	(-3.47, .49)

***p < 0.0001, **p < 0.01, p < 0.05.

Similarly, model 2 estimates, when the observation started deaths were 150 per 10,000 vehicles and there was no significant month to month variation before and immediately after the enactments of the new improved road safety policy. However, a statistical significant declining showed after its implementation by 1.96 deaths per 10,000 vehicles per month (95% CI: -3.313808 to -.6086938). On the other hand, twelve months after intervention there were 156 deaths per 10,000 vehicles (equation 1), while in the absence of this regulation the model predicted 178 deaths per 10,000 vehicles (equation 2), this implies that 12.4% of death averted by the intervention after one year of its implementation (Table 3).

However, in case of injuries, the model demonstrated that; there was no statistical significant effect of the new policy intervention on injury reduction ether immediately in the commencement or change in trend after its intervention.

## Discussion

Our finding demonstrated that, there was a statistically significant reduction of non-injury crashes and fatalities. After one year of road safety policy implementation, the rate of fatalities was 156/10,000 vehicles, showed an equivalent 12.4% reduction compared with the number of fatalities 178/10,000 vehicles, if this new road safety policy not implemented. However, there was no statistical significant reduction associated with the new road safety policy implementation and non-fatal injury reduction.

Under reporting of low severity cases and those excluded cases during data collection due to lack of necessary information, might have an effect on the overall trend. But we are not expecting similar consequences on the effectiveness of the new improved road safety policy, since the reporting system is similar before and after the intervention. In this study, we could not also take into account the potential impact of other road safety initiatives, like mass media intervention at the federal and regional level. Hence, the mass media intervention was started before the implementation of the new improved road safety policy. So we are not expecting as such a significant effect on the result. Moreover, we have considered longer pre and post-intervention period 68 months and 52 months respectively. This larger number of data points prior to the intervention helps to obtain a stable estimate of the underlying trend [12,15]. With this, we can avoid the shortcoming related to regression towards the mean and over fitting.

Abnormally high fatality rate, 156 per 10,000 vehicles was observed in this study compared with the national average, 95 death per 10,000 vehicles as reported by UN Economic Commission for Africa 2007–2008 [16]. This might be due to the high traffic volume of the road segment, as it is one of the countries import export corridor from the port Djibouti. The diverse traffic mix, including animal drawn carts frequently shares the road with high speed vehicles.

The effectiveness of policy intervention achieved by low and high income countries couldn’t be comparable for a number of reasons; in the first place the difference of road user categories frequently affected. While vehicle occupants are the most common fatally injured road users in high income countries, more than half 45–75% of all road fatalities were pedestrian in low income countries [17]. The other reason might be the level of enforcement by police officers, the availability of to date logistic supply, the safety equipment inside the vehicles and the level of police officers’ commitment.

Nineteen percent of non-injury crashes and twelve percent of deaths averted by the implementation of the new road safety policy in the study area might be due to the integration of police and transport sector at different administrative level and the establishment of a road safety committee in some parts of the region. However, 11 .2% of fatality reduction obtained in this study area after one year of intervention, still lower than the achievements of other similar low income countries, for example 30% fatality reduction in Burundi and 17% in Uganda [9,10]. The smaller effect observed in this study might be explained by, poor enforcement by traffic officers, especially related to excessive speeding. The speed limit is not respected by many of the drivers and even police officers not considered as a serious offense. Lack of speed control devices is an impediment to enforcing the law (Personal communication with traffic officers). Lack of effective enforcement for speed violators and absence of speed control devices was one of the main reason for low income countries’ poor achievement in the area of road safety [18]. In addition, there was weak enforcement on impaired driver by alcohol and khat due to the absence of a breath test and the lack of stated legal alcohol limit in the new road safety regulation.

## Conclusion

This study has shown a statistically significant reduction of non-injury crashes and fatalities after the implementation of the improved road safety regulation in the regional state. However, the problem is still grave and needs further efforts to make our road safe. Crash, injury and fatality reduction can be more promising if it is complemented by public awareness campaign, inter-sectoral collaboration and further enforcement using appropriate control devices.

## Competing interests

The authors declare that they have no competing interests.

## Authors' contributions

TA is the principal investigator and contributed to the development of research protocols, implementation of the study, and drafted the manuscript. YB and AW contributed from protocol development through writing up and assisted during data collection and commented on the draft manuscript. All authors read and approved the final manuscript.

## Pre-publication history

The pre-publication history for this paper can be accessed here:

http://www.biomedcentral.com/1471-2458/14/539/prepub
